# Iodine Excess as an Environmental Risk Factor for Autoimmune Thyroid Disease

**DOI:** 10.3390/ijms150712895

**Published:** 2014-07-21

**Authors:** Yuqian Luo, Akira Kawashima, Yuko Ishido, Aya Yoshihara, Kenzaburo Oda, Naoki Hiroi, Tetsuhide Ito, Norihisa Ishii, Koichi Suzuki

**Affiliations:** 1 Laboratory of Molecular Diagnostics, Department of Mycobacteriology, Leprosy Research Center, National Institute of Infectious Disease, 4-2-1 Aoba-cho, Higashimurayama-shi, Tokyo 189-0002, Japan; E-Mails: yuqianluo31@gmail.com (Y.L.); akirak@jichi.ac.jp (A.K.); yishido@nih.go.jp (Y.I.); aya.yoshihara@med.toho-u.ac.jp (A.Y.); kenzaburou.oda@med.toho-u.ac.jp (K.O.); 2 Department of Education Planning and Development, Faculty of Medicine, Toho University, Tokyo 143-8540, Japan; E-Mail: n-hiroi@med.toho-u.ac.jp; 3 Department of Medicine and Bioregulatory Science, Kyushu University, 3-1-1 Maidashi, Higashi-ku, Fukuoka 812-8582, Japan; E-Mail: itopapa@intmed3.med.kyushu-u.ac.jp; 4 Leprosy Research Center, National Institute of Infectious Disease, 4-2-1 Aoba-cho, Higashimurayama-shi, Tokyo 189-0002, Japan; E-Mail: norishii@nih.go.jp

**Keywords:** excess iodine, autoimmune thyroid disease, environmental toxicant, immune response, tissue injury, thyroglobulin

## Abstract

The global effort to prevent iodine deficiency disorders through iodine supplementation, such as universal salt iodization, has achieved impressive progress during the last few decades. However, iodine excess, due to extensive environmental iodine exposure in addition to poor monitoring, is currently a more frequent occurrence than iodine deficiency. Iodine excess is a precipitating environmental factor in the development of autoimmune thyroid disease. Excessive amounts of iodide have been linked to the development of autoimmune thyroiditis in humans and animals, while intrathyroidal depletion of iodine prevents disease in animal strains susceptible to severe thyroiditis. Although the mechanisms by which iodide induces thyroiditis are still unclear, several mechanisms have been proposed: (1) excess iodine induces the production of cytokines and chemokines that can recruit immunocompetent cells to the thyroid; (2) processing excess iodine in thyroid epithelial cells may result in elevated levels of oxidative stress, leading to harmful lipid oxidation and thyroid tissue injuries; and (3) iodine incorporation in the protein chain of thyroglobulin may augment the antigenicity of this molecule. This review will summarize the current knowledge regarding excess iodide as an environmental toxicant and relate it to the development of autoimmune thyroid disease.

## 1. Introduction

### 1.1. Iodine as an Essential Element for Thyroid Hormone

Iodine (symbol: I; atomic number: 53; average atomic weight: 126.9) is a trace chemical element primarily found in oceans as the highly water-soluble iodide ion (I^−^). In vertebrates, iodine is an essential element required for the production of thyroid hormone (TH). TH is highly conserved among species; its action has been well characterized in vertebrates, and increasing evidence suggests that TH also plays important roles in various invertebrates [1]. In humans, TH is important for normal growth and differentiation of cells, fetal growth, nervous system development, bone formation, reproductive tract development, *etc.* [2].

The synthesis of mammalian thyroid hormone requires the transport of I^−^ into thyroid cells. The sodium/iodide (Na^+^/I^−^) symporter (NIS), an 87-kDa transmembrane protein on the basolateral membrane of thyroid follicular cells, pumps two Na^+^ and one I^−^ from the bloodstream into cells. I^−^ is then transported across the apical membrane into the colloid of the follicular lumen by a Cl^−^/I^−^ transporter, thought to be the pendrin (PDS) protein [3,4,5,6,7]. Thyroglobulin, a large glycoprotein precursor of thyroid hormone, is also found in the colloid, following synthesis in the endoplasmic reticulum. In the colloid, the enzyme thyroid peroxidase catalyzes the oxidation of I^−^ to I_2_ and iodination of the tyrosyl residues of thyroglobulin molecules to generate monoiodotyrosine (MIT) and diiodotyrosine (DIT). Via conjugation, either two adjacent DIT particles are paired to produce thyroxine (T4) or one MIT and one DIT are paired to produce triiodothyronine (T3), which has three iodine atoms, one less iodine atom than T4 [8]. Iodinated thyroglobulin is re-absorbed by the action of thyroid stimulating hormone (TSH) into thyroid cells, where it is digested by proteases to release thyroid hormone (T4 and T3) from the backbone of its protein chain into circulation [8].

### 1.2. Global Prevention and Elimination of Iodine Deficiency

Thyroid hormone plays a central role in the intermediary metabolism of virtually all tissues and is of fundamental importance for the development of the central nervous system in the fetus and the newborn [9]. Therefore, iodine deficiency due to a lack of dietary iodine, typically seen in remote inland areas, where no marine foods are available, became a leading cause of developmental delays, mental retardation, endemic goiter and many other health problems [10]. Fortunately, iodine deficiency disorders (IDD) are a preventable public health problem with a simple and inexpensive solution. Iodine supplementation, such as universal salt iodization (USI), was introduced in order to prevent and eliminate IDD. USI is a global strategy recommended by the United Nations Children’s Fund (UNICEF), World Health Organization (WHO) in 1994 to ensure adequate dietary iodine through the addition of potassium iodate to salt. Substantial progress has been made by such global efforts to control IDD. Over the past decade, the number of iodine-deficient countries has fallen from 54 to 30; the number of iodine-sufficient countries has increased from 67 to 112; and approximately 70% of households worldwide have access to adequate iodized salt [11,12,13].

### 1.3. Iodine Excess as Another Concern

Iodine supplementation must be carefully monitored to ensure adequate iodine intake while avoiding iodine excess. WHO data show that adequate or excessive iodine intake has been observed in over 30 countries [12,13]. Investigations of these instances have identified numerous factors, including high levels of salt iodization and overlapping iodine supplementation, as well as routine consumption of particular iodine-rich foods. Risks involved in iodine excess, such as hypothyroidism, hyperthyroidism, cancers, autoimmune thyroid disease (ATD), *etc.*, have drawn more concerns than before, as iodine excess is an increasingly more frequent occurrence [11,12,13]. In the following sections, we will address iodine as an environmental toxicant and how easily excess intake can occur without accurate monitoring. We will then summarize the current knowledge about environmental iodine and proposed mechanisms by which excess iodine can contribute to the development of autoimmune thyroiditis (AIT).

## 2. Iodine: An Abundant and Easily Accessible Environmental Risk Factor for ATD

### 2.1. Excessive Iodine Exposure in the Environment

What is responsible for the excess of iodine in our bodies? Regional dietary sources that are naturally rich in iodine can contribute to iodine excess in some countries or regions. In Asian countries, such as Japan and Korea, seaweed is a popular food, especially in coastal areas. Some edible seaweed is rich in iodine [14,15] and has been identified as a unique risk factor for excess iodine in these areas. Cases of iodine excess or even iodine toxicity due to overindulging in seaweed have been reported. Investigations also indicate that this dietary pattern is associated with high morbidity of thyroid disorders, including goiter, thyroid cancers and ATD, in coastal areas of Japan [16,17,18].

In some areas of China, drinking water with high levels of iodine has been reported and identified as the key contributor to iodine excess [19,20]. Such iodine-rich drinking water has also been found in Somalia [21], Saharawi [22] and Europe [23]. The application of iodine-containing water-purification tablets is another source of excess iodine exposure from drinking water [24,25]. In the areas where residents ingest high-iodine drinking water, the iodine content of edible salt should be lowered accordingly to avoid iodine excess [26]. Moreover, due to the high iodine content in animal feed (including grass) and/or the use of iodophor cleaners for milk cans, milk and dairy products can also be rich in iodine and become a potential contributor to excess iodine in Western countries, where milk and dairy products are a major part of the diet [27,28,29,30]. Thus, when iodized salt alone is supposed to provide enough iodine, the additional routine consumption of other iodine-rich foods or drinking water can lead to chronic iodine excess in the body.

Iodine excess has been observed much more frequently since iodide supplementation by USI was initiated. Worldwide, iodinated salt, as well as processed foods containing iodized salt (e.g., bread, milk and snack foods), are the most extensive dietary sources for iodine today. However, due to the variable iodine content in edible salt, poor monitoring of production and social iodine status, salt iodine sometimes can exceed the adequate level for a particular community. A number of reports have associated high levels or overconsumption of iodized salt in food with iodine excess and thyroid disorders in Mexico [31], Somalia [32], China [26,33], Bulgaria [34], Brazil [35], Sri Lanka [36] and African countries [37,38,39,40].

Although less common, non-dietary sources of iodine sometimes contain levels that are hundreds to thousands of times higher than in the diet. Excess iodine ingestion from nutritional supplements, such as multivitamin tablets, often goes unrecognized. However, investigations in the United States showed that the actual iodine content in 60 randomly selected iodine-containing multivitamin brands varied from 11 to 610 µg per daily dose, including 15 brands with higher iodine content than was stated on the labels [41]. In addition, excess iodine ingestion from maternal nutritional supplements during pregnancy has reportedly led to congenital hypothyroidism [42].

Among iodine-rich medications, amiodarone, a drug commonly used to treat ventricular and supraventricular tachyarrhythmias, contains 37% iodine. Thus, one tablet can contain several hundred times the recommended daily intake of iodine. Moreover, amiodarone has a long half-life and easily accumulates *in vivo*. Therefore, amiodarone has been shown to be the most common medication source of excess iodine and a risk factor for medication-induced thyroid disorders [43,44]. Another common source of excess iodine in medical practices is iodinated contrast agents used for diagnostic radiology. A single dose of iodinated contrast usually contains much more iodine (hundreds of thousands of times higher) than the recommended daily dose. Iodine levels in the body will remain elevated after digestion of iodinated contrast, and it can take more than one month for iodine levels to be normalized following exposure [45]. Case reports [46,47,48,49] and the results of a large case-control study [50] support the premise that using iodinated contrast is a potential risk factor for the development of acute iodine excess and thyroid disorders. Thus, even a one-time of ingestion of any of these sources can lead to dangerous acute iodine excess. Transdermal antiseptic cleaners containing povidone-iodine (PVP-I) are also frequently used in hospitals for hand washing, gargling or wound care. Long-term use of PVP-I also presents a risk for iodine excess-induced thyroid disorders for both patients and medical workers [51,52,53].

Regardless of the iodine source, daily diet or one-time incident, exposure to excessive levels of iodine can easily occur with no awareness. While the excessive levels can be reversed, extensive recovery time may be needed to restore balance to iodine levels, a period during which thyroid functions can be significantly disturbed.

### 2.2. Excess Iodine Is a Recognized Environmental Factor for Autoimmune Thyroiditis

Although the mechanisms are not fully elucidated, excess iodine is a well-recognized environmental factor for autoimmune thyroid disease (ATD) in autoimmune-prone individuals, particularly autoimmune thyroiditis (AIT), which is characterized by lymphocytic infiltration of the thyroid gland with the development of thyroid autoantibodies and primary hypothyroidism [54]. A large body of epidemiological and clinical data from countries and regions have associated high iodine levels with the development of thyroid autoantibodies and thyroid dysfunctions, including goiter, hypothyroidism, cancers and the morbidity of AIT [35,55,56,57,58,59]. Moreover, multiple animal models, such as the BioBreeding/Worcester (BB/W) rat [60], an obese chicken strain [61,62], the Buffalo rat [63] and the non-obese diabetic (NOD) mouse [64], also suggest that excess iodine is associated with thyroid autoimmunity. The NOD.H-2h4 mouse is an autoimmune thyroiditis-prone animal model that is used extensively for the study of iodine-induced AIT. The administration of iodine can significantly enhance and accelerate, in a dose-dependent manner, the incidence of AIT, its onset, the degree of lymphocytic infiltration and the severity of damage to thyroid follicular structures [65,66].

Conversely, iodine also has a minor role in the treatment for hyperthyroidism. Saturated iodine solutions are sometimes used to treat Graves’ disease (which is usually accompanied by hyperthyroidism), another major ATD in addition to AIT. The treatment is contingent on the fact that a high concentration of iodine acutely inhibits thyroid hormone secretion within hours and temporarily inhibits iodine organification, as well as thyroid hormone synthesis, presumably due to the phenomenon of the Wolff-Chaikoff effect [67,68]. However, the mechanism underlying the use of iodine to treat Graves’ disease may involve more than the negative feedback effect on iodine organification and thyroid hormone synthesis. *In vivo* and *in vitro* evidence shows that even a short period of administrating a high concentration of iodine could reduce the expression of major histocompatibility complex (MHC) class I and class II (molecules that mediate antigen presentation and are thought to be important factors in the development of autoimmune disease) in the thyrocytes of Graves’ patients [69]. The exact mechanism is unknown, but probably involves nuclear factor κB-mediated gene expression [16]. Iodine depletion has also been associated with increased MHC expression in nontoxic goiters [70], indicating another potential effect of iodine deficiency.

As the pathologies of AIT and Graves’ disease are significantly different and most evidence links iodine excess to autoimmune thyroiditis, the next sections mainly focus on mechanisms that have been proposed to explain the association of excessive iodine with AIT.

## 3. Mechanisms Involved in Iodine-Induced Autoimmune Thyroiditis

### 3.1. Stimulation of Lymphocytic Response in Thyroid by Excess Iodine

Iodine-induced thyroiditis has been observed in autoimmune-prone animals and is characterized by lymphocytic infiltrations in the thyroid, increased cytokine secretion, induced expression of MHC class II on thyrocytes and elevated thyroid autoantibody titers. In NOD.H-2h4 mice, CD4^+^ T cells are the first to appear in the thyroid shortly after iodine treatment, followed by the sequential appearance of CD8^+^ T-cells, macrophages and B-cells [65,71]. Interleukin (IL)-12 and interferon (IFN)-γ-positive cells enter the thyroid early in the focal accumulation of infiltrating cells. MHC class II expression is clearly induced by iodine treatment in thyrocytes, especially in those near the focal lymphocytic infiltrations [71]. Thyroid autoantibody titers are significantly upregulated along with the progression of lymphocytic infiltration [72]. Both thyroiditis and autoantibody generation can be abolished by depleting the populations of CD4^+^ T-cells, CD8^+^ T-cells or B-cells [73,74,75], indicating a critical role of lymphocytic infiltration in the development of iodine-induced AIT. Similarly, lymphocytic infiltrations form in autoimmune-prone BB rats following iodine treatment, a process that begins with an increase of MHC class II-positive dendritic cells and a clustering of these cells with T-cells, B-cells and macrophages in the thyroid. The presence of lymphocytic infiltration has been positively correlated to thyroid autoantibody titers in the serum of these BB rats [76].

Intercellular adhesion molecule (ICAM)-1 is an important regulator of the immune response. It promotes cell-to-cell interaction and provides intense signals to the immune system to initiate the homing of lymphocytes to an inflamed site. Iodine treatment increases the percentage of ICAM-1-expressing thyrocytes in NOD.H-2h4 mice and in primary cultured mouse thyrocytes [77]. In primary human thyroid follicular cells from Graves’ patients, ICAM-1, monocyte-derived neutrophil chemotactic factor (MDNCF) and other chemokines, such as chemokine (C-C motif) ligand 2 (CCL2), chemokine (C-X-C motif) ligand 8 (CXCL8), and CXCL14, that can attract immunocompetent cells to the thyroid were increased by high concentrations of iodine [78,79]. Immunohistochemical staining of Graves’ thyroid sections revealed that the chemokines were expressed primarily in thyrocytes in addition to lymphocytes [78]. These iodine-induced cytokines and chemokines secreted by both lymphocytes and thyrocytes likely induce and promote iodine-induced lymphocytic infiltration in autoimmune-prone thyroids.

Further cytokine analyses have shown that IFN-γ, IL-4 and IL-17, the effector cytokines of Th_1_, Th_2_ and Th_17_ respectively, are expressed in the thyroids of NOD.H-2h4 mice with iodine-induced thyroiditis [65,80]. IFN-γ was maximally expressed shortly after iodine treatment, while IL-4 was maximally expressed only when lymphocytic infiltration was maximally severe and chronic [65]. Ablation of IFN-γ, or IL-17, but not IL-4, in NOD.H2h4 mice demonstrated resistance to both thyroiditis and the generation of anti-thyroglobulin autoantibodies, indicating that both Th_1_ and Th_17_ cells are the T-cell subsets critical for the pathogenesis of iodine-induced AIT in these mice [80,81].

CD4^+^/CD25^+^/Foxp3^+^ regulatory T-cells and Foxp3 gene expression were reduced in mice with iodine-induced thyroiditis [72], and the number of regulatory T-cells was negatively related to the severity of thyroiditis [82]. Iodine-induced thyroiditis was inhibited, at least in part, by the increased number of regulatory T-cells in NOD.H-2h4 mice expressing transgenic transforming growth factor (TGF)-β on thyrocytes [83]; while depletion of regulatory T-cells by anti-CD25 antibody before iodine treatment significantly exacerbated iodine-induced thyroiditis and increased anti-thyroglobulin antibody titers [84]. These studies suggested that regulatory T-cells play an important role in the negative regulation of iodine-induced autoimmune thyroiditis.

In summary, iodine induces cytokine and chemokine-mediated lymphocytic infiltration in the thyroids of autoimmune-prone individuals, which is critical for the generation of thyroid autoantibodies and thyroiditis. Therefore, the next question is: how does iodine induce this lymphocytic infiltration in the thyroid? Excess iodine-induced oxidative cell injury has been proposed as a potential trigger for this lymphocytic response.

### 3.2. Induction of Oxidative Thyroid Tissue Injury by Excess Iodine

In thyroid, follicular cell injury, apoptosis and necrosis that precede lymphocytic infiltration in the thyroid are considered the initial events in, and prerequisites for, the development of iodine-induced autoimmune thyroiditis [85,86]. Activation of several death receptor-mediated signaling pathways, including Fas ligand, tumor necrosis factor-related apoptosis-inducing ligand (TRAIL) and B-cell lymphoma (Bcl)-2-associated X protein, is consistently observed in thyrocytes after iodine treatment [86].

It was shown that high-dose iodine induced thyrocyte injury in both the wild-type and obese strain (OS) that has a genetic background prone to spontaneous autoimmune thyroiditis. However, significant and sustained lymphocytic infiltration composed of CD4^+^ T-cells, CD^+^8 T-cells, B-cells and macrophages was only observed in OS chickens following iodine-induced cell injury. Pre-treatment with the antioxidant drug, ethoxyquin, completely prevented both thyrocyte injury and the following lymphocytic infiltration induced by iodine [87]. This study suggests that excess iodine can induce oxidative stress-related thyrocyte injury in individuals, although whether this cell injury leads to lymphocytic infiltration will depend on the additional effects of genetic factors. *In vitro* studies have shown that incubation with iodine significantly increases intracellular and extracellular reactive oxygen species (ROS) in thyrocytes derived from NOD.H-2h4 mice. It also enhanced ICAM-1 expression, which is involved in directing lymphocytes homing to the thyroid. Inhibition of a subunit protein of nicotinamide adenine dinucleotide phosphate (NADPH) oxidase p67^phox^ (an enzyme that catalyzes the generation of ROS products) by its inhibitor, diphenyleneiodonium, prior to iodine treatment abolishes both iodine-induced ROS generation and ICAM-1 expression in NOD.H-2h4 thyrocytes, indicating that iodine-induced ICAM-1 expression is associated with ROS levels in NOD.H-2h4 thyrocytes [88,89].

Under basal conditions, thyroid epithelial cells produce moderate amounts of ROS that are required for iodine oxidation and organification during thyroid hormone synthesis. ROS are not necessarily harmful, because they are continuously balanced by the process of hormone synthesis and the endogenous antioxidant system. Unbalanced redundant ROS can cause cell injury via oxidation of cell components (such as lipid oxidation), and severe cell injury beyond repair can induce apoptosis or necrosis. Additionally, oxidative cleavage of human thyroglobulin protein can expose immunodominant thyroglobulin peptides that are recognized by autoantibodies in the serum of patients with AIT [90,91], indicating the role of oxidative cleavage in the generation of immunogenic and pathogenic thyroglobulin peptides. It has been reported that, in addition to increased ROS levels, high doses of iodine increase malondialdehyde (a lipid peroxide), lipofuscin (a product of lipid peroxidation that may be symptomatic of damage to the membrane, mitochondria and lysosomes) and accumulation of peroxisomes and secondary lysosomes (organelles that contain various oxidative enzymes and oxidants) [92,93,94,95], indicating that active lipid peroxidation occurred after high dose iodine administration. An *in vitro* study using primary normal human thyrocytes demonstrated that a direct acute toxic effect of excess iodine characterized by ultrastructural lesions (such as cytoplasmic fragment desquamation, endoplasmic reticulum vesiculation, *etc.*), the accumulation of lipofuscin in secondary lysosomes and necrosis can be prevented by inhibitors of iodine trapping or organification [96].

Administration of iodine can promote the involution of hyperplastic thyroids induced either by a low iodine diet or by an anti-thyroidal medication, propylthiouracil. When such hyperplastic thyroids received a moderate amount of iodine, the involution was revealed as only a mode of cell deletion via apoptosis without detectable necrosis or inflammation [97]. In contrast, in high dose iodine-treated involuting thyroids, apoptosis was accompanied by abundant necrosis and lymphocytic infiltrations, which could be abolished by inhibition of iodine oxidation and organification, but not by inhibition of vasoconstriction [94,97]. Moreover, lipofuscin accumulation in thyrocytes was always associated with necrosis and only seen in excess iodine-treated involuting thyroids [97]. Chronic treatment with high levels of iodine in autoimmune-prone goitrous mice can induce symptoms similar to Hashimoto’s thyroiditis, including destruction of thyroid follicles, infiltrations composed of CD4^+^ T-cells, CD8^+^ T-cells and B-cells and thyroid autoantibodies in the plasma [85]. These studies suggest that the toxic effect of excess iodine on thyroid cells may be due to immoderate lipid peroxidation, induced by an excessive number of ROS that generate during the process of oxidation and organification of excess iodine.

Recent studies suggest that immune responses can be induced not only by pathogen-associated molecular patterns (PAMPs), but also by danger (or damage)-associated molecular patterns (DAMPs) [98]. PAMPs, the most well-recognized triggers for immune response, refer to various substances derived from pathogens, including bacteria, viruses, parasites and fungi, which induce immune response through the mediation of pattern recognition receptors, like Toll-like receptors. In contrast to exogenous PAMPs, DAMPs are endogenous intracellular and extracellular molecules (such as genomic DNA fragments, heat shock proteins, collagen and hyaluronic acid) released from sterile cells damaged by various stimuli, such as ischemia/reperfusion injury, trauma and oxidative stress [98]. Our group has reported that genomic DNA fragments released from injured rat thyroid cells can be recognized by extrachromosomal histone H2B, leading to the activation of both innate and acquired immune responses in thyroid cells and the suppression of thyroid function, suggesting that thyroid tissue injury is a potential trigger for autoimmune reaction and the induction of thyroid dysfunction [99]. Moreover, stimulation of double-stranded RNA (a typical DAMP) significantly increased the expression of interferon-responsive genes, cytokines and chemokines and suppressed thyroid functional gene expression in cultured human thyroid cells [100]. Comparable to the immune response activation stimulated by PAMPs or DAMPs, treatment with high concentrations of iodine also significantly increased the gene expression of various cytokines in cultured human thyroid cells [100]. The totality of the evidence shows that it is conceivable that oxidative cell injury, caused by the processing of excessive iodine, results in the generation of DAMPs that induce immune response in thyroid cells. However, a high concentration iodine-induced immune-related gene expression profile differs in part from those induced by typical DAMPs [100], indicating that high concentration iodine may also have a direct effect on the activation of immune response in thyroid cells.

### 3.3. Influence of Iodine on Thyroglobulin Auto-Antigenicity

MHC class II expression can be induced in thyrocytes following the stimulation of cell injury caused by excess iodine, thus facilitating the presentation of auto-antigens. Lymphocytes simultaneously begin migrating to injured, inflamed sites in the thyroid, where they will meet the major thyroid auto-antigen, thyroglobulin, the most common target of thyroid autoantibodies in AIT. The high immunogenicity of thyroglobulin is thought to be related to its large size, abundance, glycosylation and polymorphisms [101]. As iodination can alter the structure and affect proteolytic degradation of thyroglobulin [102,103], it is reasonable to presume a role for iodine in modifying the auto-antigenicity of thyroglobulin. Numerous studies have been devoted to exploring the role of iodine in the auto-antigenicity of thyroglobulin that is associated with AIT.

Cornel strain (CS) chickens, a strain genetically prone to AIT, fed a high iodine diet produce high iodine thyroglobulin (HI-Tg) with at least 60 iodine atoms per molecule, while those fed a low iodine diet produce low iodine thyroglobulin (LI-Tg) with less than 13 atoms per molecule. HI-Tg-immunized CS chickens produce abundant serum antibodies that react well with HI-Tg and thyroid hormones, but only weakly with LI-Tg. LI-Tg-immunized CS chickens produce very few antibodies to LI-Tg and thyroid hormones, but a modest amount of antibodies to HI-Tg [61]. This study suggests that high iodine diets can indeed lead to the production of thyroglobulin with high iodine content and that the anti-thyroglobulin autoantibody has a higher affinity to HI-Tg than to LI-Tg. Ebner *et al.* [104] showed in susceptible BB/W rats that only iodine-rich thyroglobulin, and not non-iodinated thyroglobulin, could induce lymphocytic thyroiditis. Moreover, iodine content in thyroglobulin has been associated with its self-recognition by T-cells. Champion *et al.* [105] showed that two clonotypically distinct, thyroglobulin-specific, MHC class II-restricted T-cell populations recognized thyroglobulin only when it was sufficiently iodinated, while non-iodinated thyroglobulin could not induce significant thyroid lesions. Sufficiently iodinated thyroglobulin, but not poorly iodinated thyroglobulin, induced a proliferative response in a cloned T-cell line, 2D11, from a diseased NOD.H2h4 mouse [106]. Re-iodination *in vitro* could re-establish the immunogenicity of these non-iodinated or poorly iodinated thyroglobulins [104,106]. Rasooly *et al.* [64] were the first to report that recognition by human T-cells of human thyroglobulin depends upon its iodine content. They found that lymphocytes from the blood of either autoimmune thyroiditis patients or healthy people did not display a proliferative response upon stimulation of non-iodinated thyroglobulin, while iodinated thyroglobulin produced significant proliferation of lymphocytes from both groups.

In the past decade, immunogenic peptides recognized by anti-thyroglobulin autoantibodies in serum from ATD patients or associated with ATD in mouse models have been mapped within the 2749 amino acid sequence of thyroglobulin [107,108]. Based on iodine involvement, these thyroglobulin peptides can be divided into three categories: (1) hormonogenic site-containing peptides; (2) iodotyrosyl residue-containing peptides; and (3) iodine-free peptides [109]. The effect of iodine on the immunogenicity of these peptides is not necessarily positive or direct, but rather, complex and depends on the nature of each peptide. Hormonogenic site-containing peptides are 12mer thyroglobulin peptides containing a T4 site. T4(2553) (T4 positioned at amino acid 2553) was the first reported thyroiditis-associated thyroglobulin peptide [73,110]. Removal of the four iodine atoms from T4(2553) did not abolish its ability to activate primed T-cells to transfer thyroiditis in CBA mice [111]. However, in other studies, iodine on the thyroxine ring structure was critical for the recognition of T4-containing peptides by two clonotypically-distinct, thyroglobulin-specific, MHC class II-restricted cloned T-cell populations [112]. These seemingly contradictory results suggest that iodine may be required for the immunogenicity of T4-containing peptides only if its antigen presentation is mediated exclusively by MHC class II molecules. In fact, studies have suggested that T4-containing peptides are not major thyroglobulin epitopes that are presented by MHC class II, as they are moderately stimulatory for thyroglobulin-primed MHC class II-deficient T-cells [113]. Thus, although it appears that iodine *per se* is not required for the immunogenicity of T4-containing peptides, it may modify or reinforce their immunogenicity.

Among iodotyrosyl residue-containing peptides, three peptides (p117, p304 and p1931) have been reported to be immunogenic and pathogenic only in their iodinated forms, presumably through the conformation alteration induced by iodination, thus facilitating either peptide binding to MHC or T-cell recognition of the peptide [114]. The effects of iodination on the immunogenicity of some other iodotyrosyl residue-containing peptides are variable, depending on the position of the iodine atom and the property of the lymphocyte populations in a particular reaction. Iodination of immunogenic thyroglobulin peptide p179 (amino acids 179–194) has different effects on a panel of T-cell hybridoma clones: some clones require iodination for activation; some react only with non-iodinated p179, while others can react with both [115]. Thus, T-cells from different hybridoma clones may exhibit different tolerances to iodine content in a particular thyroglobulin peptide, making it more difficult to predict the effect of iodine when T-cells consist of polyclonal populations [115]. Furthermore, enhanced iodination of thyroglobulin selectively facilitates the processing and presentation of cryptic pathogenic peptides, even though the peptide itself is iodine-free. Dai *et al.* [116] demonstrated that lymphocytes from SJL mice challenged with highly iodinated thyroglobulin displayed a stronger proliferative response upon the stimulation of an iodine-free thyroglobulin peptide p2495, but not p2694, *in vitro*, while lymphocytes from SJL mice challenged with normal thyroglobulin did not respond to either. Thus, the effect of the iodine atom on the auto-antigenicity and pathogenicity of thyroglobulin greatly depends on the particular peptide that is modified by iodine, either directly or indirectly.

## 4. Conclusions

Iodine excess is now recognized as an environmental risk factor for the development of ATD in humans and animals. Several underlying mechanisms may explain its action: redundant ROS generated during trapping, oxidation and organification of excessive iodine in thyrocytes (likely due to a defect in the iodine processing machinery) can lead to elevated oxidative stress and consequential oxidative cell damage. This damage may stimulate thyrocytes as danger (or damage)-associated molecular patterns to produce and secrete cytokines and chemokines, thus recruiting lymphocytes to the thyroid [98,99,100], where they meet major thyroid auto-antigens, including thyroglobulin. Modification by excessive iodine may alter the conformation of the thyroglobulin molecule to facilitate its antigen presentation by professional antigen presenting cells, as well as MHC-expressing thyrocytes and its recognition by T-cells. Thus, excessive iodine will eventually lead to pathological intolerance to thyroid auto-antigens and the development of thyroiditis.

It is usually considered to be safe to ingest a relatively large amount of iodine from the diet, as most people are highly tolerant to iodine. However, the elderly population, pregnant women, fetuses, neonates and those with pre-existing goiter or iodine deficiency are more susceptible to excess iodine-induced disorders, including ATD. Thus, iodine is indeed an environmental risk factor for the development of ATD, especially in susceptible individuals. Therefore, the iodine status in a community should be carefully monitored to provide accurate data for establishing a social strategy for nutrient ingestion.
